# Supplementary material for: Orkambi® and amplifier co‐therapy improves function from a rare CFTR mutation in gene‐edited cells and patient tissue
Source: EMBO Mol Med. 2017 Jun 30;9(9):1224–43. doi: 10.15252/emmm.201607137 (PMC5582412; doi:10.15252/emmm.201607137)
Supplement: Supplementary file 1 — Expanded View Figures PDF [file EMMM-9-1224-s001.pdf]

Expanded View Figures

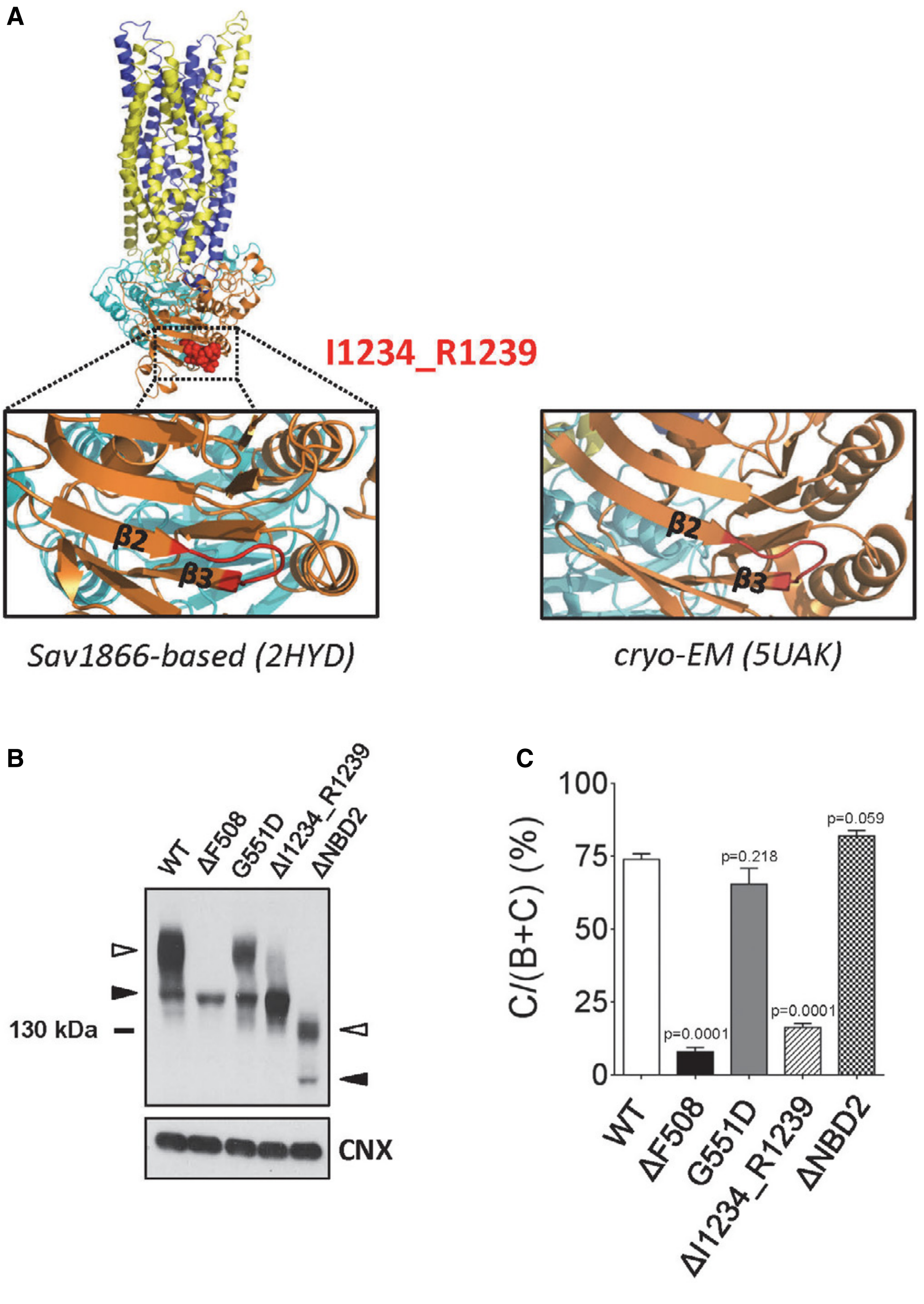

Figure EV1. Location of I1234_R1239 in the WT-CFTR homology model and relative protein expression levels between clinically relevant CFTR variants.

A WT-CFTR homology model (left side; Dalton *et al*, 2012) showing the predicted secondary structure of I1234_R1239 in the open-channel state, and the WT-CFTR cryo-electron microscopy-derived structure (right side; Liu *et al*, 2017) showing the secondary structure of I1234_R1239 in the closed-channel state. MSD1, blue; NBD1, cyan; R-domain, omitted from model; MSD2, yellow; NBD2, orange; I1234_R1239 sequence, red.

B, C Immunoblots (B) and quantitative analysis (C) showing steady-state expression levels of ΔI1234_R1239-CFTR and other CFTR mutants (ΔF508-CFTR, G551D-CFTR, ΔNBD2-CFTR). Band B, black arrowhead; band C, white arrowhead. Mean ± SEM, n = 4 biological replicates with statistical significance of difference relative to WT-CFTR indicated as P-values using unpaired t-tests.

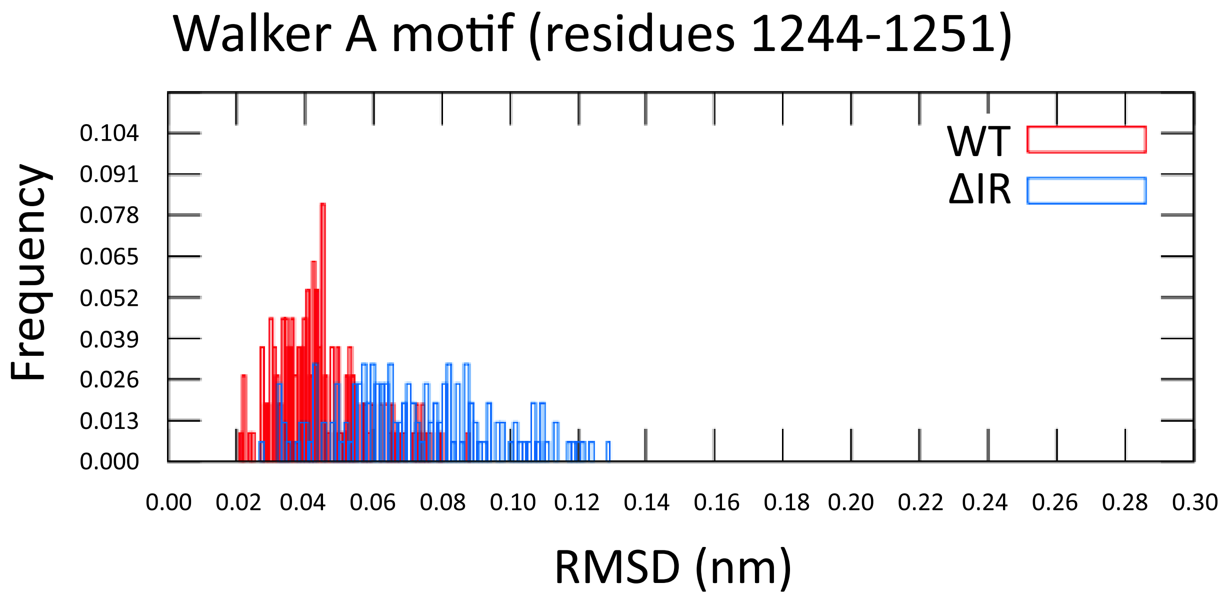

Figure EV2. Structural heterogeneity of Walker A in WT- and ΔI1234_R1239-NBD2.
Distribution of pairwise RMSDs for final states of the backbone atoms of the Walker A motif (residues 1,244–1,251) in WT-NBD2 (red) and ΔI1234_R1239-NBD2 (blue).

© 2017 The Authors

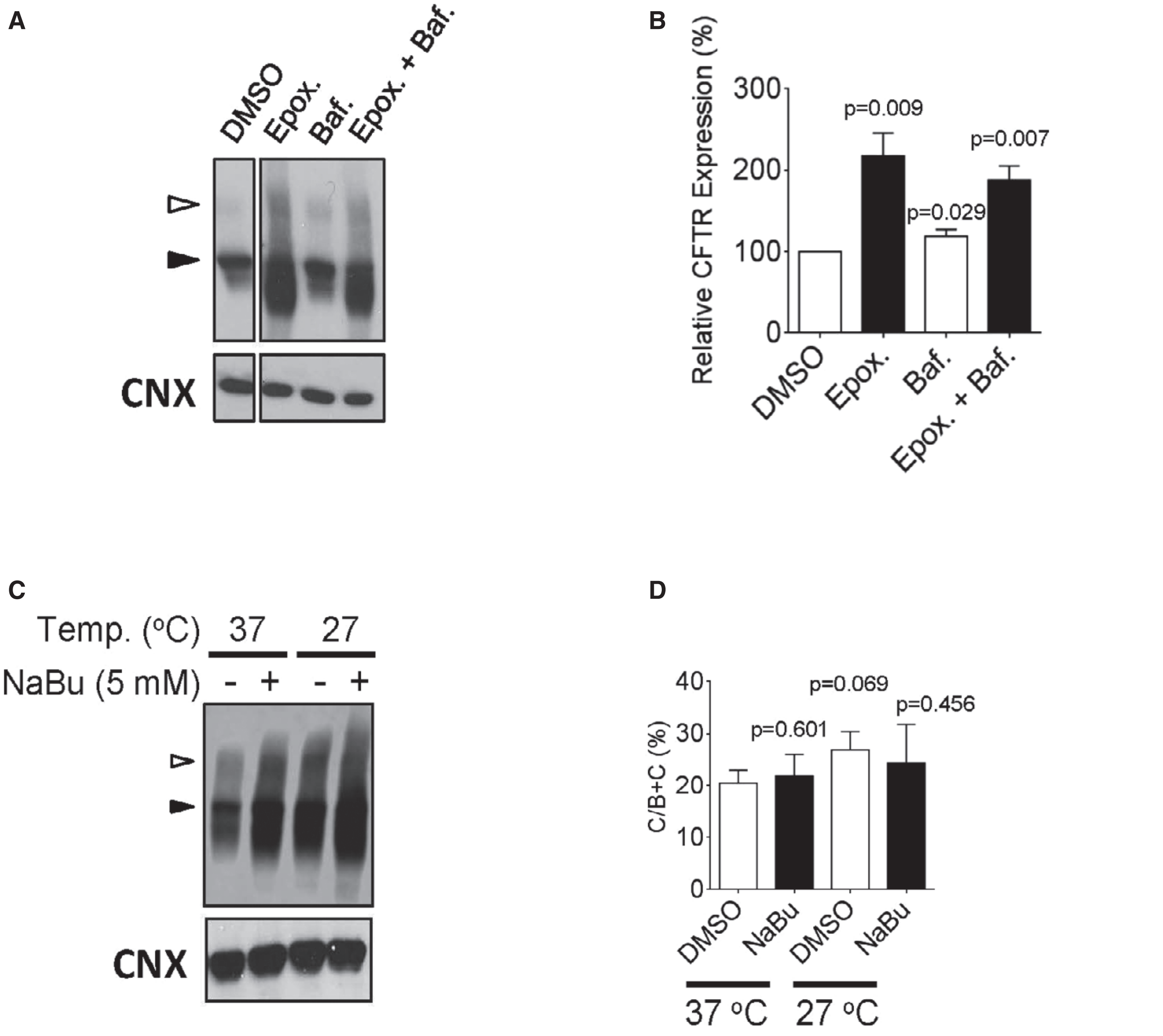

Figure EV3. ΔI1234_R1239-CFTR is degraded by the proteasome, and abundance, but not maturation is enhanced by a transcriptional modulator and low temperature incubation.

A, B Immunoblots (A) and quantitation (B) of ΔI1234_R1239-CFTR, transiently transfected into HEK-293 cells following treatments with epoxomicin (proteasome inhibitor) or bafilomycin A1 (lysosomal degradation inhibitor). Band B, black arrowhead; band C, white arrowhead. Mean ± SEM, n = 3 biological replicates with statistical significance of difference relative to DMSO indicated as P-values using unpaired t-tests.

C Immunoblots of ΔI1234_R1239-CFTR following treatments with sodium butyrate (NaBu, transcriptional modulator) or low temperature incubation (27°C). Band B, black arrowhead; band C, white arrowhead.

D Quantitation of ΔI1234_R1239-CFTR rescue % (band C/bands B + C) after low temperature and/or sodium butyrate (NaBu) treatment. Mean ± SEM, n = 3 biological replicates with statistical significance of difference relative to DMSO (37°C) indicated as P-values using unpaired t-tests.

© 2017 The Authors

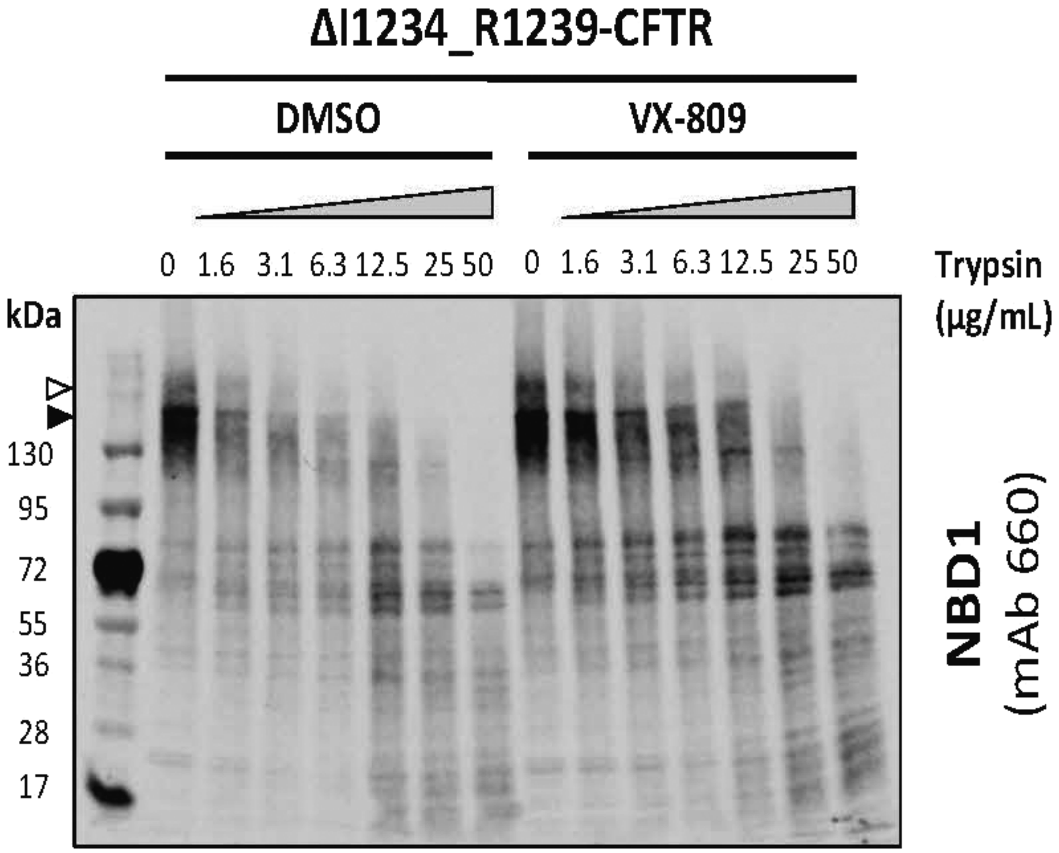

Figure EV4. Proteolytic digestion and probing of amino-terminal fragments of ΔI1234_R1239-CFTR in HEK-293 cells using mAb 660 in the absence (DMSO) or presence of VX-809.

The trypsin digestion profile of ΔI1234_R1239-CFTR as studied using anti-NBD1 mAb 660 is comparable to the profile using a distinct anti-NBD1 antibody: L12B4. Band B, black arrowhead; band C, white arrowhead.

 © 2017 The Authors

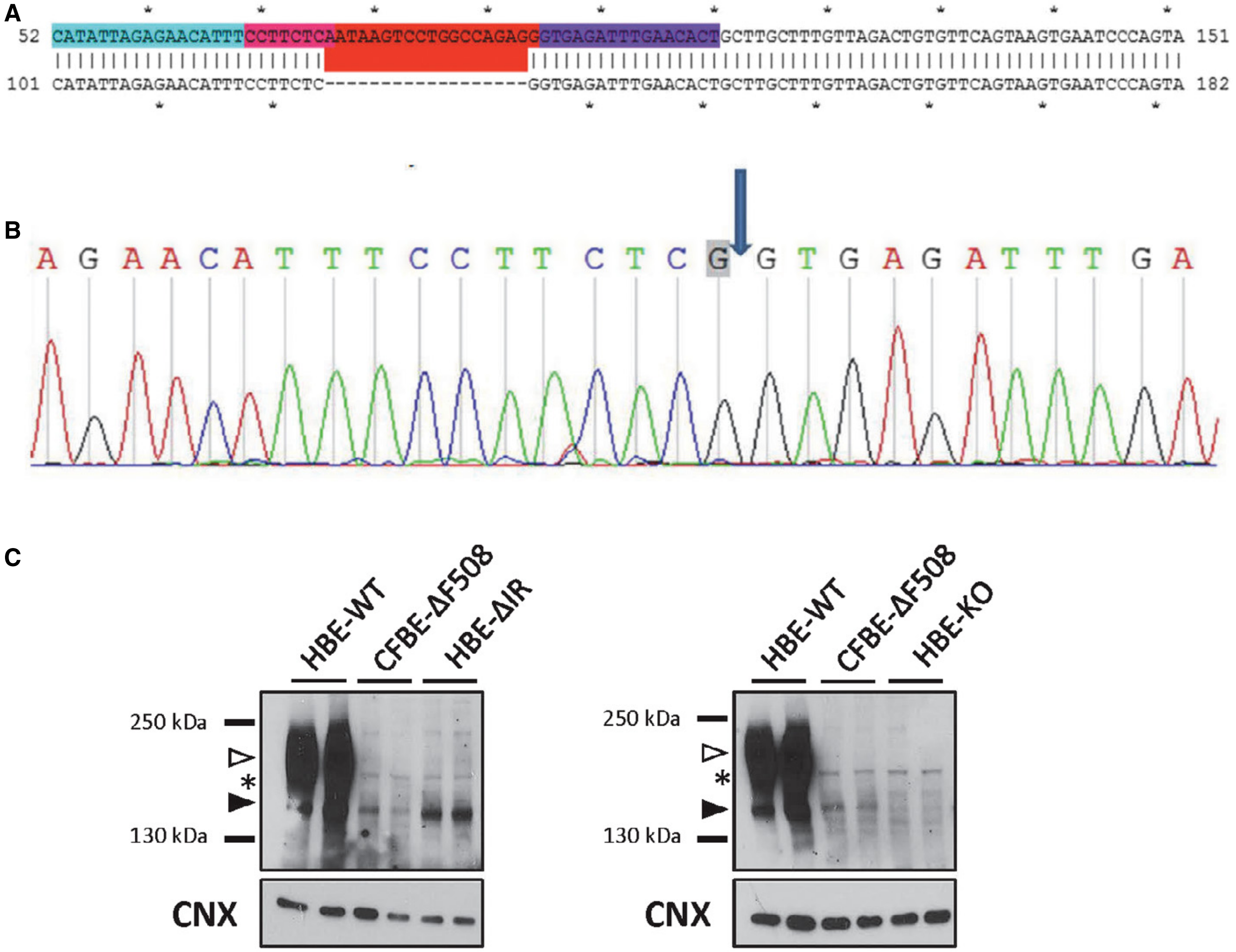

Figure EV5. CRISPR/Cas9 editing of *CFTR* alleles to generate an HBE cell line constitutively expressing ΔI1234_R1239-CFTR.

A Engineering strategy to delete 18 nt (red) in both alleles of the *CFTR* gene of HBE cells expressing WT-CFTR.

B Sequencing confirms deletion of 18 nt and presence of engineered splice site donor, C>G (gray box). The arrow indicates the boundary between adjacent exons.

C Immunoblots of HBE cell lines endogenously expressing WT-CFTR, ΔF508-CFTR and ΔI1234_R1239-CFTR (left panel), as well as comparison to an HBE cell line in which *CFTR* alleles were disrupted (i.e., 'knocked out', HBE-KO). Band B, black arrowhead; band C, white arrowhead; *, non-specific mAb artifact.

© 2017 The Authors

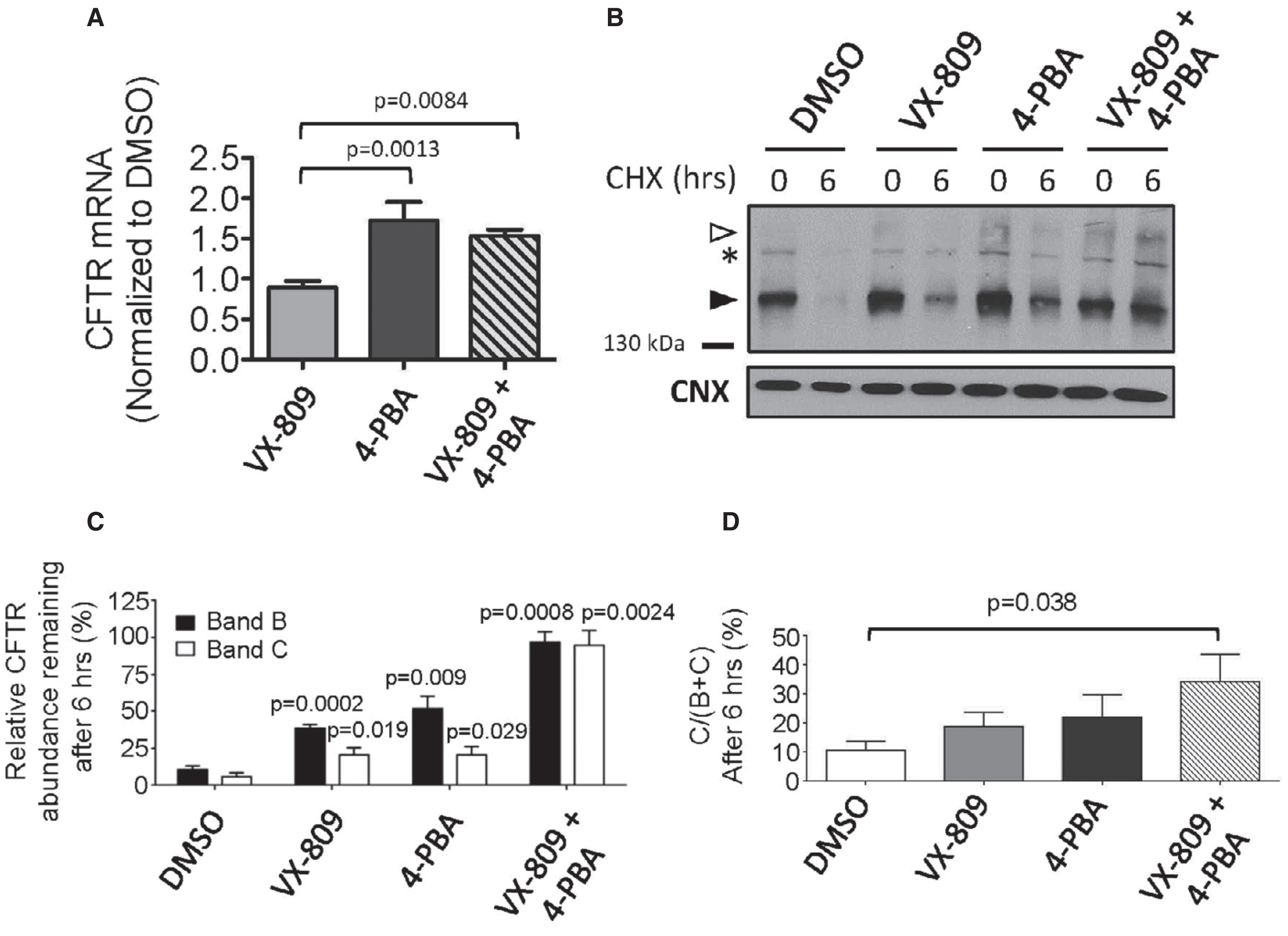

Figure EV6. VX-809 and 4-PBA lead to enhanced conformational maturation of ΔI1234_R1239-CFTR in a CRISPR/Cas9-edited cell line and in patient-derived nasal epithelial cells.

A Quantitation of relative *CFTR* mRNA expression levels in HBE-ΔIR cells following pharmacological treatments. Mean ± SEM, $n = 3$ for each intervention and statistical significance tested using one-way ANOVA with Bonferroni's multiple comparisons test.

B Immunoblot showing results of 6-h cycloheximide chase (to inhibit protein translation) after pre-treatment of CRISPR/Cas9-edited cell line bearing ΔI1234_R1239-CFTR with vehicle (DMSO), VX-809 (3 μM), 4-PBA (1 mM), or VX-809 + 4-PBA. CNX was used as a loading control. Band B, black arrowhead; band C, white arrowhead; *, non-specific mAb artifact.

C Quantitation of expression of ΔI1234_R1239-CFTR in HBE cells following treatments with VX-809, transcriptional modulator (HDAC inhibitor) 4-phenylbutyrate (4-PBA), or VX-809 + 4-PBA before (0 h) or 6 h after cycloheximide (CHX) treatment. Mean ± SEM, $n = 3$ for each intervention and statistical significance of comparison to DMSO controls indicated above each bar. Statistical significance tested using two-way ANOVA with Tukey's multiple comparisons test.

D Quantitation of mutant protein maturation (band C/bands B + C) after treatments described above. Mean ± SEM, $n = 3$ for each intervention and statistical significance of comparison to DMSO assessed using paired *t*-tests.

 © 2017 The Authors

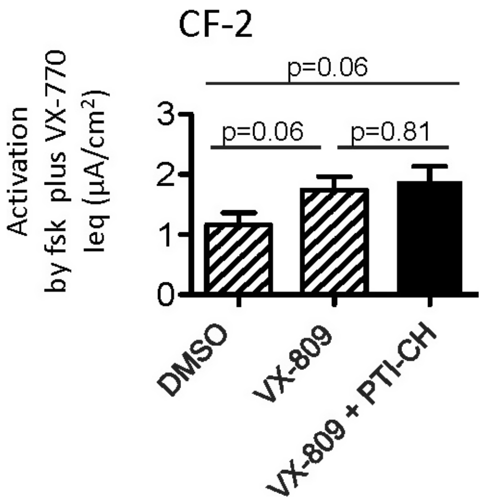

Figure EV7. Summary of forskolin- and VX-770-dependent responses in Ussing chamber studies of nasal cultures from subject CF-2.

Bars represent mean and SEM of five biological replicates (different nasal cell seedings). Statistical significance assessed using paired *t*-tests. The combination of VX-809 (3 μM) + PTI-CH (1 μM; black bar) did not enhance forskolin and VX-770 activated Ieq relative to VX-809 alone (hatched bar corresponding to primary nasal culture data shown in Fig 6E).
