# Supplementary material for: Orkambi® and amplifier co‐therapy improves function from a rare CFTR mutation in gene‐edited cells and patient tissue
Source: EMBO Mol Med. 2017 Jun 30;9(9):1224–43. doi: 10.15252/emmm.201607137 (PMC5582412; doi:10.15252/emmm.201607137)
Supplement: Supplementary file 2 — Table EV1 [file EMMM-9-1224-s002.doc]

| **CF-1** | | | | **CF-2** | | | |
| --- | --- | --- | --- | --- | --- | --- | --- |
| **Genetic Analysis** | **Genetic Variant** | **Interpretation** | **T-tract status** | **Genetic Analysis** | **Genetic Variant** | **Interpretation** | **T-tract status** |
| ***CFTR* gene sequencing** | c.1408G>A (p.Met470Val) | Reported polymorphism*- homozygous |  | ***CFTR* gene sequencing** | c.1408G>A (p.Met470Val) | Reported polymorphism*- homozygous |  |
| ***CFTR* gene sequencing** | c.3700A>G (p.Ile1234Val) | Reported mutation*- homozygous |  | ***CFTR* gene sequencing** | c.3700A>G (p.Ile1234Val) | Reported mutation*- homozygous |  |
| ***CFTR* gene sequencing** | c.869+11C>T | Reported polymorphism*- homozygous |  | ***CFTR* gene sequencing** | c.869+11C>T | Reported polymorphism*- homozygous |  |
| ***CFTR* gene sequencing** | c.744-33GATT[6]+[6] | Reported polymorphism*- homozygous |  | ***CFTR* gene sequencing** | c.744-33GATT[6]+[6] | Reported polymorphism*- homozygous |  |
| **Recurrent mutation panel** | None detected |  |  | **Recurrent mutation panel** | None detected |  |  |
| ***CFTR* gene dosage** | normal |  |  | ***CFTR* gene dosage** | normal |  |  |
| **Genomic DNA analysis of poly-T-tract** |  |  | 9T/9T | **Genomic DNA analysis of poly-T-tract** |  |  | 9T/9T |

**Table EV1 - Identical *CFTR* mutations and polymorphisms detected in both CF Subjects.**

Both CF affected individuals tested by multiple-PCR analysis for 39 recurrent *CFTR* mutations, by gene dosage using MLPA and by direct sequence analysis of the coding and flanking region of the *CFTR* gene [NCBI reference sequence NC_000007.13; NM_000492.3].

Recurrent mutations tested: G542X; G85E; R334W; Y122X; R560T; 3905insT; 3876delA; 2789+5G>A; G551D; R117H; A455E; S549R; R347H; 1078delT; 1717-1G>A; 3120+1G>A; S1255X; A559T; Y1092X; M1101K; 2307insA; 2183AA>G; 1898+5G>T.
